# Supplementary material for: Per1/Per2 Disruption Reduces Testosterone Synthesis and Impairs Fertility in Elderly Male Mice
Source: Int J Mol Sci. 2022 Jul 2;23(13):7399. doi: 10.3390/ijms23137399 (PMC9266724; doi:10.3390/ijms23137399)
Supplement: Supplementary file 1 [file ijms-23-07399-s001.zip › ijms-1754549-SI.pdf]

Supplementary Data for

Per1/*Per2* disruption reduces testosterone synthesis and impairs fertility in elderly male mice

Qinrui Liu [1†], Hu Wang [2†], Hualin Wang[3], Na Li [4], Ruyi He [5,*] and Zhiguo Liu [6,*]

1 School of life science and technology, Wuhan Polytechnic University, Wuhan, China; 524669367@qq.com
2 School of life science and technology, Wuhan Polytechnic University, Wuhan, China; 592887102 @qq.com
3 School of life science and technology, Wuhan Polytechnic University, Wuhan, China; wanghualin313@163.com
4 School of life science and technology, Wuhan Polytechnic University, Wuhan, China; lina81518@qq.com
5 School of life science and technology, Wuhan Polytechnic University, Wuhan, China; hry.1226@qq.com
6 School of life science and technology, Wuhan Polytechnic University, Wuhan, China; zhiguo_l @126.com
* Correspondence: zhiguo_l@126.com; hexiaoyi@outlook.com
† These authors contributed equally to this work

This PDF file includes:

Supplementary Tables S1 to S4

Supplementary Figures S1 to S7

Table S1 Feed formulation of experimental diets

Ingredient	Proportion
Corn starch	40%
Soybean meal	20%
Fish Meal	7%
Wheat flour	23%
Dry yeast	4.5%
Calcium hydrophosphate	2%
Mountain flour	1.5%
Vit-min premix	0.3%
Mineral premix	1.7%

Table S2 qPCR primer of this study

Gene	Forward Primer(5'- 3')	Reverse Primer(5'- 3')
β-actin	CTACCTCATGAAGATCCTGACC	CACAGCTTCTCTTTGATGTCAC
Star	TCAACTGGAAGCAACACTCTAT	ATCTTACTTAGCACTTCGTCCC
Hsd3b1	AAAGGTACCCAGAACCTATTGG	CTGTATGGGTATGGATCAGACC
Hsd3b6	AAGTTCTTCAGACCAGAAACCA	TACTGGGTGTCAAGAATGTCTC
Cyp11a1	AGTATTATCAGAGGCCCATTGG	AACATCTGGTAGACAGCATTGA
Cyp17a1	GAGGTGAAGAGGAAGATCCAAA	ATACGAAGCACTTCTCGGATAG
Cyp19a1	TCATGAAGCACAGTCACTACAT	AAACTTCCACCATTCGAACAAG
Hsd17b3	CGCCGATGAGTTTGTTAAAGAA	GGATCCGGTTCAGAATTATTGC
Cyp21a1	CAAGATGTGGTGGTGCTAAATT	GCCTTCCACATGAGAGAGTAAT
Cyp11b1	GAATGTGTATCGAGAGCTGGCAGAG	TTGATGTCGTGTCAGTGCTTCCAG
Ugt1a6a	ATCCAAAGACTCGGGCATTCATCAC	ATCATCACCATCGGAACTCCATTGC
Ugt1a7c	ATCCAAAGACTCGGGCATTCATCAC	ATCATCACCATCGGAACTCCATTGC

Table S3 Changes in the ratio of DKO/ WT-related differential genes in KEGG pathways

KEGG Pathway	Gene Symbol	Description	log2FC	P value
Steroid hormone biosynthesis	*Cyp17a1*	cytochrome P450, family 17, subfamily a, polypeptide 1	0.538 ↓	$5.87*10^{-17}$
	Cyp11a1	cytochrome P450, family 11, subfamily a, polypeptide 1	0.487 ↓	$1.67*10^{-13}$
	Cyp21a1	cytochrome P450, family 21, subfamily a, polypeptide 1	0.788 ↓	0.028
	Ugt1a6a	UDP glucuronosyltransferase 1 family, polypeptide A6A	1.220 ↓	$3.00*10^{-14}$
	Ugt1a10	UDP glycosyltransferase 1 family, polypeptide A10	5.246 ↑	0.00087
	Ugt1a7c	UDP glucuronosyltransferase 1 family, polypeptide A7C	0.608 ↓	0.00095
	Cyp21a1	cytochrome P450, family 21, subfamily a, polypeptide 1	0.788 ↓	0.00119
	Hsd17b3	hydroxysteroid (17-beta) dehydrogenase 3	0.383 ↓	0.0028
	Sult1e1	sulfotransferase family 1E, member 1	0.511 ↓	0.0054
	Cyp2e1	cytochrome P450, family 2, subfamily e, polypeptide 1	2.086 ↑	0.0075
	Hsd3b1	hydroxy-delta-5-steroid dehydrogenase, 3 beta- and steroid delta-isomerase 1	0.182 ↓	0.0223<br>0.0456
	Cyp3a41a	cytochrome P450, family 3, subfamily a, polypeptide 41A	3.002 ↓	0.0499
Cortisol synthesis and secretion	*Cyp17a1*	cytochrome P450, family 17, subfamily a, polypeptide 1	0.538 ↓	$5.87*10^{-17}$
	Cyp11a1	cytochrome P450, family 11, subfamily a, polypeptide 1	0.485 ↓	$1.67*10^{-13}$
	Star	steroidogenic acute regulatory protein	0.541 ↓	$7.88*10^{-9}$
	Plcb4	phospholipase C, beta 4	0.427 ↓	$2.11*10^{-17}$
	Cyp21a1	cytochrome P450, family 21, subfamily a, polypeptide 1	0.788 ↓	0.000280

	Agt	angiotensinogen (serpin peptidase inhibitor, clade A, member 8)	0.208 ↓	0.0103
	Creb3l1	cAMP responsive element binding protein 3-like 1	0.360 ↓	0.0157
	Cacna1h	calcium channel, voltage-dependent, T type, alpha 1H subunit	0.158 ↑	0.0177
	Scarb1	scavenger receptor class B, member 1	0.124 ↓	0.0279
	Hsd3b1	hydroxy-delta-5-steroid dehydrogenase, 3 beta- and steroid delta-isomerase 1	0.182 ↓	0.0456
	Ldlr	low density lipoprotein receptor	0.439 ↓	0.0477
Aldosterone synthesis and secretion	*Cyp11a1*	cytochrome P450, family 11, subfamily a, polypeptide 1	-0.485 ↓	$1.67*10^{-13}$
	Star	steroidogenic acute regulatory protein	-0.541 ↓	$7.88*10^{-}$
	Plcb4	phospholipase C, beta 4	-0.427 ↓	$^{09}2.11*10^{-}$
	Cyp21a1	cytochrome P450, family 21, subfamily a, polypeptide 1	-0.788 ↓	^{04} 0.00280
	Agt	angiotensinogen (serpin peptidase inhibitor, clade A, member 8)	-0.208 ↓	0.0103
	Creb3l1	cAMP-responsive element binding protein 3-like 1	-0.360 ↓	0.0157
	Cacna1h	calcium channel, voltage-dependent, T type, alpha 1H subunit	0.158 ↑	0.0177
	Scarb1	scavenger receptor class B, member 1	-0.124 ↓	
	Prkcg	protein kinase C, gamma	0.701 ↑	0.0279
	Atp1a2	ATPase, Na+/K+ transporting, alpha 2 polypeptide	0.157 ↑	0.0327 0.0430
	Dagla	diacylglycerol lipase, alpha	0.522 ↑	
	Hsd3b1	hydroxy-delta-5-steroid dehydrogenase, 3 beta- and steroid delta-isomerase 1	-0.182 ↓	0.0431 0.0456
	Ldlr	low-density lipoprotein receptor	-0.439 ↓	0.0477

Table S4 plasma of T, ALD, CORT, DOC, A4, P, LH levels in WT and DKO mice

	WT (n=8)	DKO (n=8)	*P* value
Testosterone (ng/mL)	0.3371 ± 0.05291	0.116 ± 0.006902	0.0060**
Aldosterone (ng/mL)	0.1937 ± 0.03848	0.1759 ± 0.03639	0.7419
Corticosterone (ng/mL)	93.11 ± 4.814	105.1 ± 8.333	0.2322
11-Deoxycorticosterone (ng/mL)	1.803 ± 0.2599	3.098 ± 0.9717	0.2189
Androstendione (ng/mL)	0.09029 ± 0.07627	0.1531 ± 0.08578	0.6131
Progesterone (ng/mL)	0.6761 ± 0.08387	0.7625 ± 0.2151	0.6851
Luteinizing hormone (mIU/mL)	1.98 ± 0.6555	1.02 ± 0.4048	0.2444

Values are mean ± SD； * P<0.05,** P<0.01

SUPPLEMENTARY FIGURES

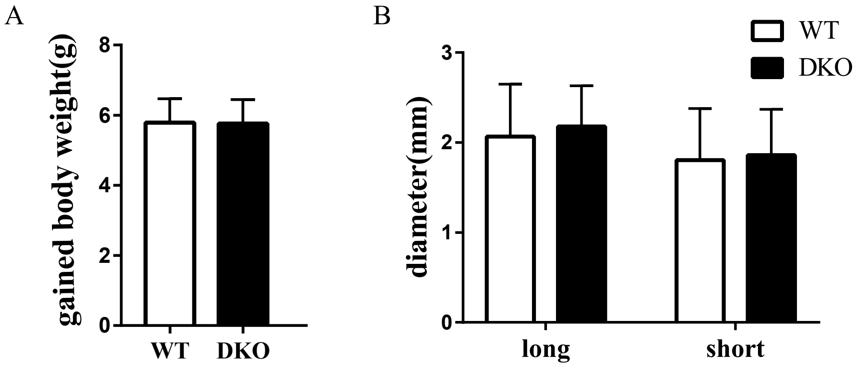

Figure S1 Testicular morphology data of two types. DKO and WT male mice (A) weight gain (15-month-age - 9-month-age weight). (B) The testicular minor and major axis

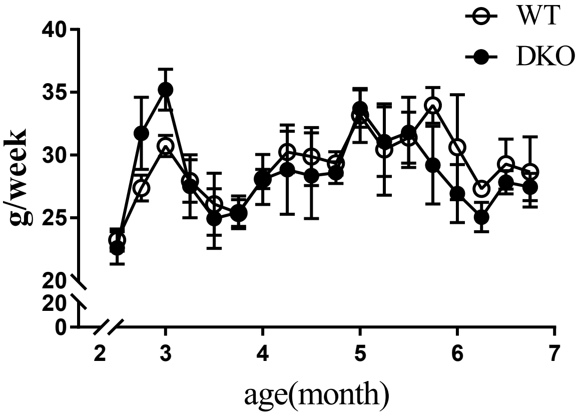

Figure S2 Feed intake of mice per week of WT and DKO mice from 2-month-age to 7-month-age.

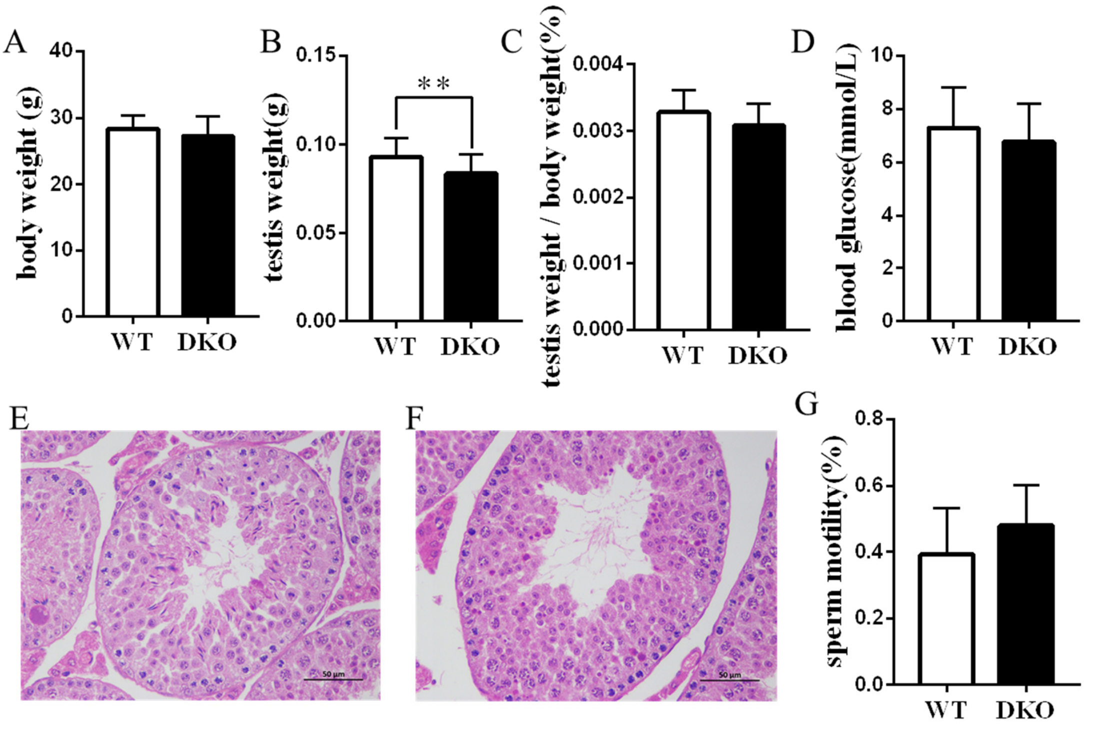

Figure S3. Physiological effect indices and HE staining results of 9-month DKO and WT mice. (A) Bodyweight growth curve; (B) Testicular weight; (C) Ratio of testis weight/body weight; (D) Fasting blood glucose levels; (E) HE staining of WT testis; (F) HE staining of DKO testis; (G) Sperm motility; （n =10; * p <0.05;** p <0.01

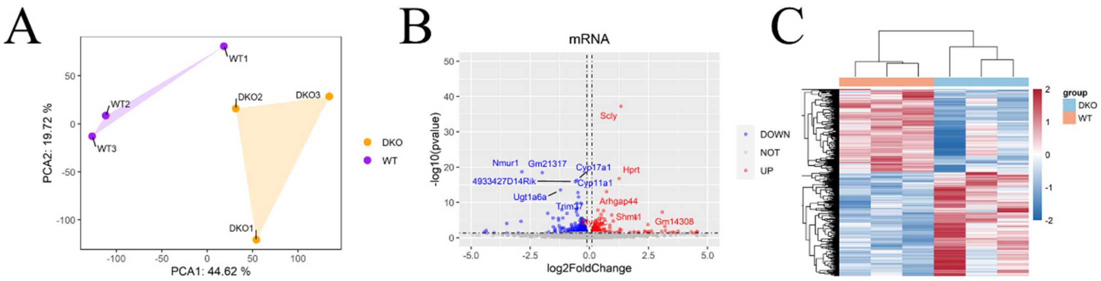

Figure S4 Differentially expressed testis RNA between DKO and WT mice (|log2 fold change| >0.1, P.value < 0.05). Compared with normal samples, 532 mRNA were upregulated and 425 mRNA were downregulated in DKO mice. (A) Principal component analysis (PCA) of DKO samples and WT samples; (B) is a volcano map for differentially expressed mRNAs. Red stands for up-regulation, blue stands for down-regulations, and gray stands for intermediate in volcanoes. Each point represents a gene. (C) is the heatmap for differentially expressed mRNAs. Red stands for DKO male mice, and blue stands for WT male mice.

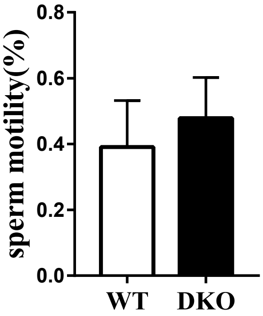

Figure S5 Sperm motility of WT and DKO mice in 9-month-age.

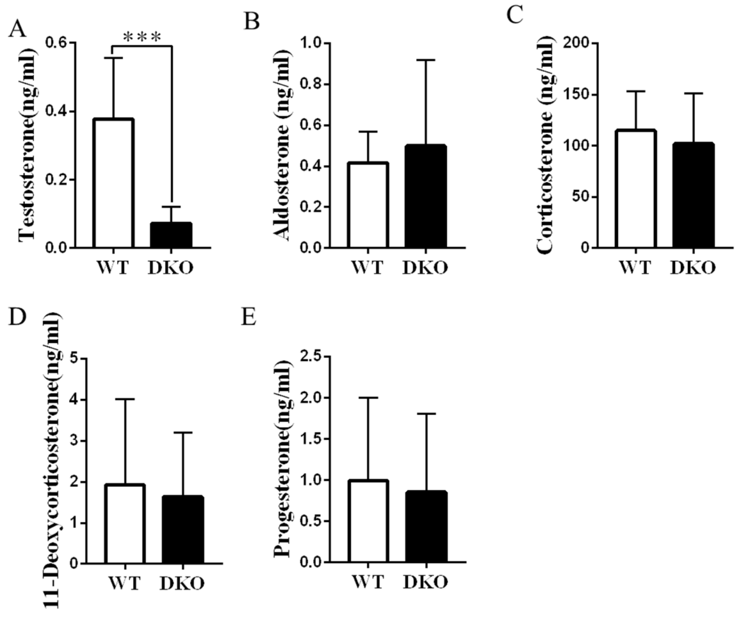

Figure S6. Plasma steroid hormone levels in 9-month-age WT and DKO male mice. (A)-(G): T, ALD, CORT, DOC, P (n =6). * p <0.05; ** p <0.01; *** p <0.001.

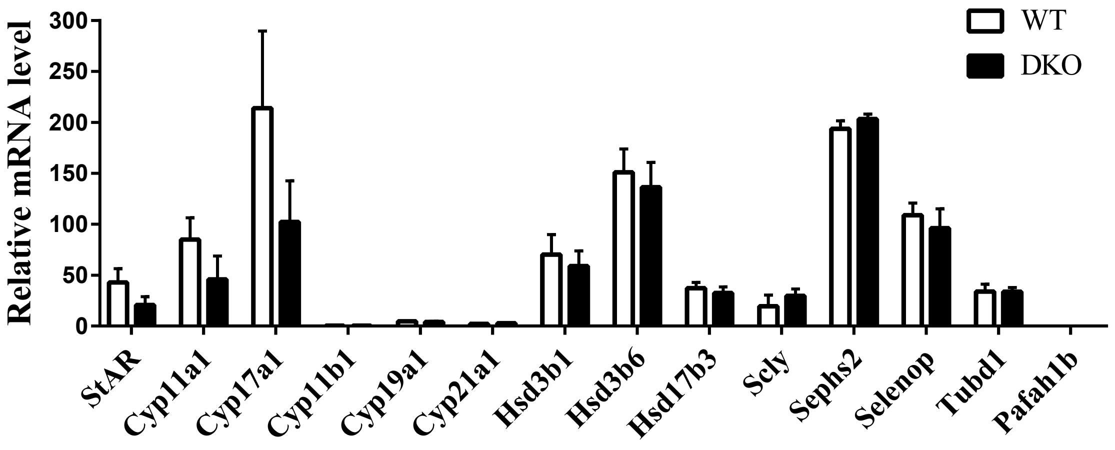

Figure S7. Expression of genes associated with testicular steroid biosynthesis, tubulin dynamics and Seleno-compound metabolic loci.
